# Reduced tolerance to abiotic stress in transgenic *Arabidopsis* overexpressing a *Capsicum annuum* multiprotein bridging factor 1

**DOI:** 10.1186/1471-2229-14-138

**Published:** 2014-05-20

**Authors:** Wei-Li Guo, Ru-Gang Chen, Xiao-Hua Du, Zhen Zhang, Yan-Xu Yin, Zhen-Hui Gong, Guang-Yin Wang

**Affiliations:** 1 College of Horticulture, Northwest A&F University, Yangling, Shaanxi, P R China; 2 State Key Laboratory of Crop Stress Biology in Arid Areas, Northwest A&F University, Yangling, Shaanxi, P R China; 3 School of Horticulture Landscape Architecture, Henan Institute of Science and Technology, Xinxiang, Henan, P R China

**Keywords:** *Capsicum annuum* L, Cold stress, Salt stress, *CaMBF1*, *Arabidopsis*

## Abstract

**Background:**

The pepper fruit is the second most consumed vegetable worldwide. However, low temperature affects the vegetative development and reproduction of the pepper, resulting in economic losses. To identify cold-related genes regulated by abscisic acid (ABA) in pepper seedlings, cDNA representational difference analysis was previously performed using a suppression subtractive hybridization method. One of the genes cloned from the subtraction was homologous to *Solanum tuberosum MBF1* (*StMBF1*) encoding the coactivator multiprotein bridging factor 1. Here, we have characterized this *StMBF1* homolog (named *CaMBF1*) from *Capsicum annuum* and investigated its role in abiotic stress tolerance.

**Results:**

Tissue expression profile analysis using quantitative RT-PCR showed that *CaMBF1* was expressed in all tested tissues, and high-level expression was detected in the flowers and seeds. The expression of *CaMBF1* in pepper seedlings was dramatically suppressed by exogenously supplied salicylic acid, high salt, osmotic and heavy metal stresses. Constitutive overexpression of *CaMBF1* in *Arabidopsis* aggravated the visible symptoms of leaf damage and the electrolyte leakage of cell damage caused by cold stress in seedlings. Furthermore, the expression of *RD29A*, *ERD15*, *KIN1*, and *RD22* in the transgenic plants was lower than that in the wild-type plants. On the other hand, seed germination, cotyledon greening and lateral root formation were more severely influenced by salt stress in transgenic lines compared with wild-type plants, indicating that *CaMBF1*-overexpressing *Arabidopsis* plants were hypersensitive to salt stress.

**Conclusions:**

Overexpression of *CaMBF1* in *Arabidopsis* displayed reduced tolerance to cold and high salt stress during seed germination and post-germination stages. *CaMBF1* transgenic *Arabidopsis* may reduce stress tolerance by downregulating stress-responsive genes to aggravate the leaf damage caused by cold stress. *CaMBF1* may be useful for genetic engineering of novel pepper cultivars in the future.

## Background

Transcriptional regulatory proteins play a central role in the expression of genomic information during complex biological processes in all organisms. Among these proteins, transcriptional co-activators are key components of eukaryotic gene expression by interacting with both transcription factors and/or other regulatory elements and the basal transcription machinery [1,2]. Multiprotein bridging factor 1 (*MBF1*), a transcriptional co-activator, enhances transcription of its target genes by bridging the general factor TBP (TATA box Binding Protein) and specific transcription factors bound to their target promoters in eukaryotes such as yeast [3], *Drosophila*[4] and *Arabidopsis*[5].

*MBF1*-type genes (*SlER24* and *StMBF1*) encode functional transcriptional co-activators as demonstrated by their capacity to complement the yeast *mbf1* mutant [6,7]. Fusion of tomato *SlER24* to EAR (Amphiphilic Repression) in the MicroTom cultivar induced a delay of seed germination, but had no obvious effect on plant growth [6]. Moreover, it was reported that the *StMBF1* gene in potato was induced by pathogen attack, oxidative stress, wounding and in response to salicylic acid (SA) treatment [7,8]. Direct evidence of the involvement of *MBF1* in plant responses to environmental stresses was obtained by enhancing tolerance to heat and osmotic stresses in transgenic *Arabidopsis* lines expressing the *AtMBF1c* gene and more recently *AtMBF1a*, without growth retardation [9,10]. These data indicate that *MBF1*-like genes can be associated with a variety of developmental processes in plants such as environmental stress tolerance. To date, there are very few data on the significance of *MBF1* in cold stress tolerance.

Pepper (*Capsicum annuum* L.) is a member of the Solanaceae family, and an important vegetable and spice crop valued for its aroma, taste, pungency and flavor. The pepper fruit is the second most consumed vegetable around the world [11]. Different types of peppers, including chili, mild and sweet peppers are cultivated worldwide. Low temperature is one of the most important abiotic factors limiting the growth, development and geographical distribution of plants [12]. Pepper plants originate from tropical regions and are very sensitive to low temperature, which affects their vegetative development and reproduction, resulting in economic losses [13-15]. As part of production and fruit quality improvement, we are interested in investigating plant defense mechanisms to improve resistance to environmental stresses. In our previous report, we showed that exogenous application of ABA increased the tolerance of pepper seedlings to chilling-induced oxidative damage, mainly by enhancing the activity of antioxidant enzymes and expression of related genes [16]. Furthermore, ABA-mediated candidate genes associated with chilling stress have been fully characterized in pepper plants using a suppression subtractive hybridization (SSH) method [17]. One of the genes cloned from the reverse subtraction was homologous to *Solanum tuberosum MBF1* (*StMBF1*) encoding the coactivator multiprotein bridging factor 1. Expression of this *MBF1* homologue was highly induced by cold stress, whereas ABA-pretreatment decreased its expression in pepper seedlings subjected to cold stress. However, the function of this gene involved in the defense response to chilling stress remains to be elucidated.

In this study, based on the above-mentioned expressed sequence tag (EST) from the reverse SSH library that enriched the up-regulated expressed genes responding to chilling stress, we have functionally characterized the homolog of *StMBF1* in pepper (designated as *CaMBF1*). The results of this study suggest that *CaMBF1* transcript in pepper seedlings can be suppressed by SA, salt, osmotic and heavy metal stresses. Overexpression of *CaMBF1* in *Arabidopsis* displayed reduced tolerance to cold and high salt stress.

## Results

### Isolation of the *CaMBF1* cDNA clone and sequence analysis

**Figure 1:**
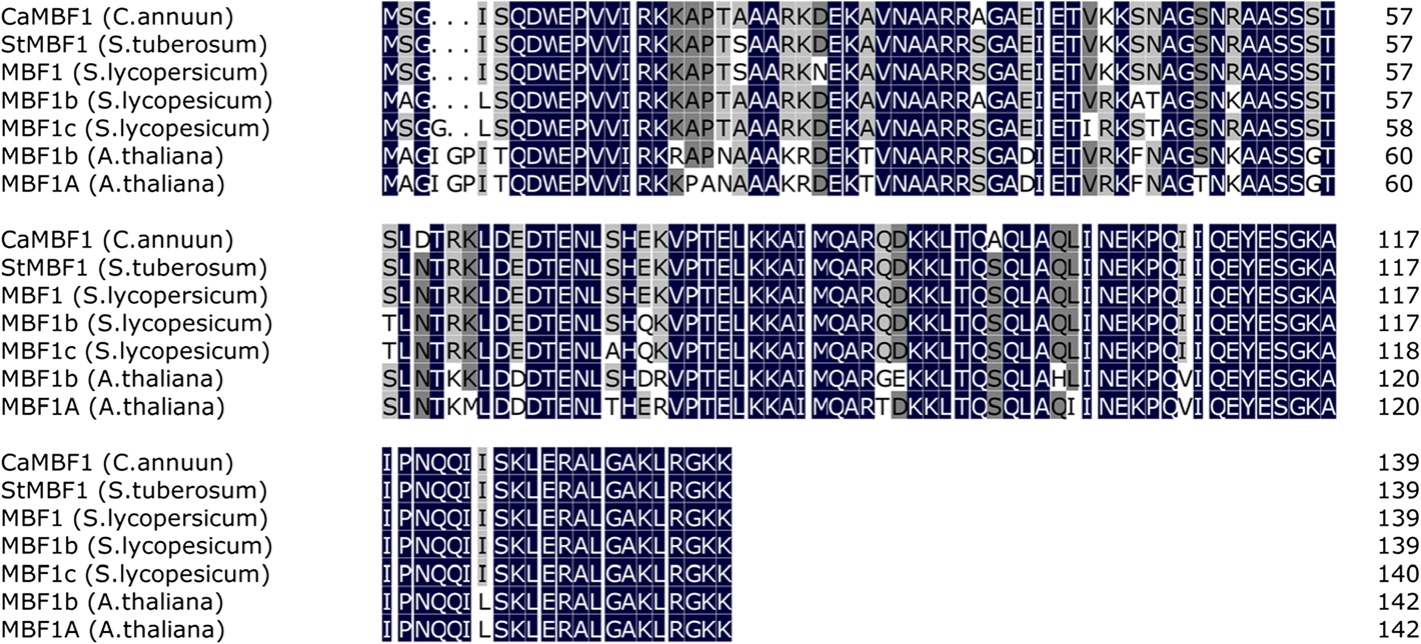
**Alignment of deduced amino acid sequences of *CaMBF1 *and other MBF proteins.** StMBF1 (AAF81108.1) from *Solanum tuberosum*, MBF1 (NP_001234341.1), MBF1b (XP_004251896.1), MBF1c (ABG29114.1) from *Solanum lycopersicum* and MBF1A (NP_565981.1), MBF1b (NP_191427.1) from *Arabidopsis thaliana*. Conserved residues are shaded in black, dark grey shading indicates similar residues in at least six out of the seven sequences, and light grey shading indicates similar residues in four to five out of the seven sequences.

A differential screening of a cold-related pepper seedling cDNA library, using PCR-amplified subtracted and control probes, was performed previously [17]. One of the isolated clones exhibited 80% identity at the nucleotide level to *StMBF1* from *Solanum tuberosum*[8]. A full-length clone of this homologue was obtained by a homology-based candidate gene method, including the complete open reading frame. The gene was named *CaMBF1* and submitted to GenBank with the Accession Number JX402927. The size of the *CaMBF1* clone was 648 bp, comprising an open reading frame of 420 bp (139 amino acids). The predicted polypeptide was basic, with a pI of 9.86 and a molecular mass of 15.3 kDa. An alignment of the deduced amino acid sequence of *CaMBF1* with other homologous sequences is presented in Figure 1. At the amino acid level, *CaMBF1* showed a high degree of conservation with known genes of other plant species: *Solanum tuberosum* (*StMBF1*, 95% identity) [8], and *Arabidopsis thaliana* (*AtMBF1b*, 80% identity; *AtMBF1a*, 79% identity) [10].

### Expression of *CaMBF1* in pepper seedlings is severely suppressed by stress and SA treatments

**Figure 2:**
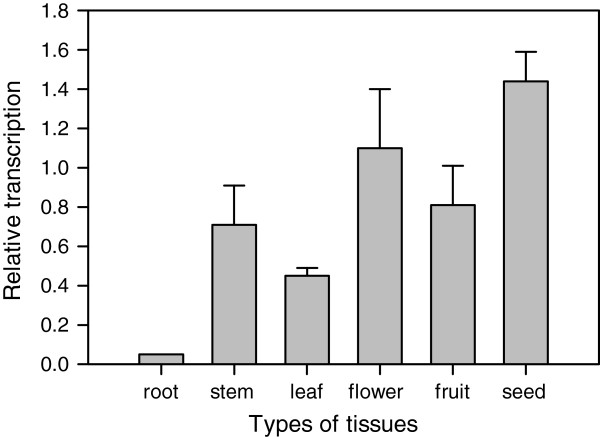
**Tissue specific expression of *CaMBF1 *in pepper seedlings.** Pepper *UBI-3* gene (GenBank No. AY486137.1) was used as an internal control for normalization of different cDNA samples.

**Figure 3:**
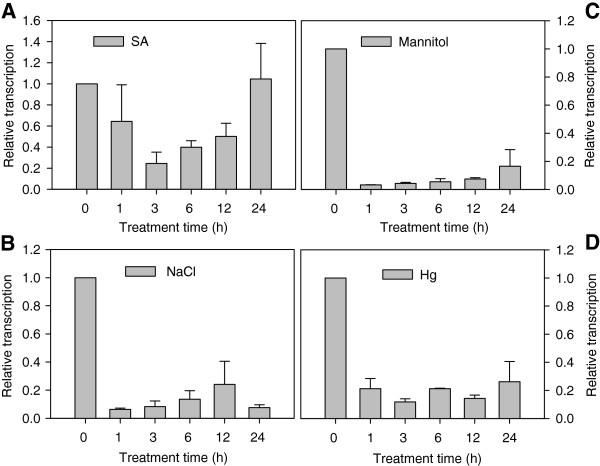
**Analysis of *CaMBF1 *expression profiles in pepper seedlings in response to different stress treatments.** The pepper seedlings were sprayed with 5 mM SA solution **(A)**; the pepper seedlings were exposed to salt stress (300 mM NaCl) **(B)**, osmotic stress (300 mM mannitol) **(C)** and heavy metal (300 μM Hg) **(D)** for the indicated times (0, 1, 3, 6, 12 and 24 h). Pepper *UBI-3* gene (GenBank No. AY486137.1) was used as an internal control for normalization of different cDNA samples. The expression level of *CaMBF1* at 0 h was used as control (quantities of calibrator) and was assumed as 1. Error bars represent standard error of means based on three independent reactions.

A number of *MBF1* genes were found to be differentially induced by abiotic stress [10,18,19]. Therefore, we suspected that the *CaMBF1* gene may be involved in stress signaling pathways and were interested in its possible function in stress responses. As a first step toward functional analysis, we examined the expression pattern of *CaMBF1* in pepper plants using qRT-PCR analysis. This analysis revealed that the *CaMBF1* gene was expressed ubiquitously in all developmental stages of plants and in all tested organs, including root, stem, leaf, flower, fruit and seed (Figure 2). High-level expression was detected in flower and seed, although expression level in root was rather low. As shown in Figure 3, *CaMBF1* expression was dramatically decreased by several stress conditions, including 5 mM SA, high salt (300 mM NaCl), osmotic stress (300 mM mannitol), and heavy metal (300 μM Hg). Rapid and robust down-regulation of *CaMBF1* transcript was observed at 1 h after salt, osmotic and heavy metal treatments, which decreased to 0.06-fold, 0.03-fold and 0.12-fold, respectively. In contrast, a slight reduction of *CaMBF1* transcript was found during 12 h of SA treatment and followed by an increase to the initial level (Figure 3A).

### Reduced tolerance of *CaMBF1*-overexpressing *Arabidopsis* plants to cold stress

**Figure 4:**
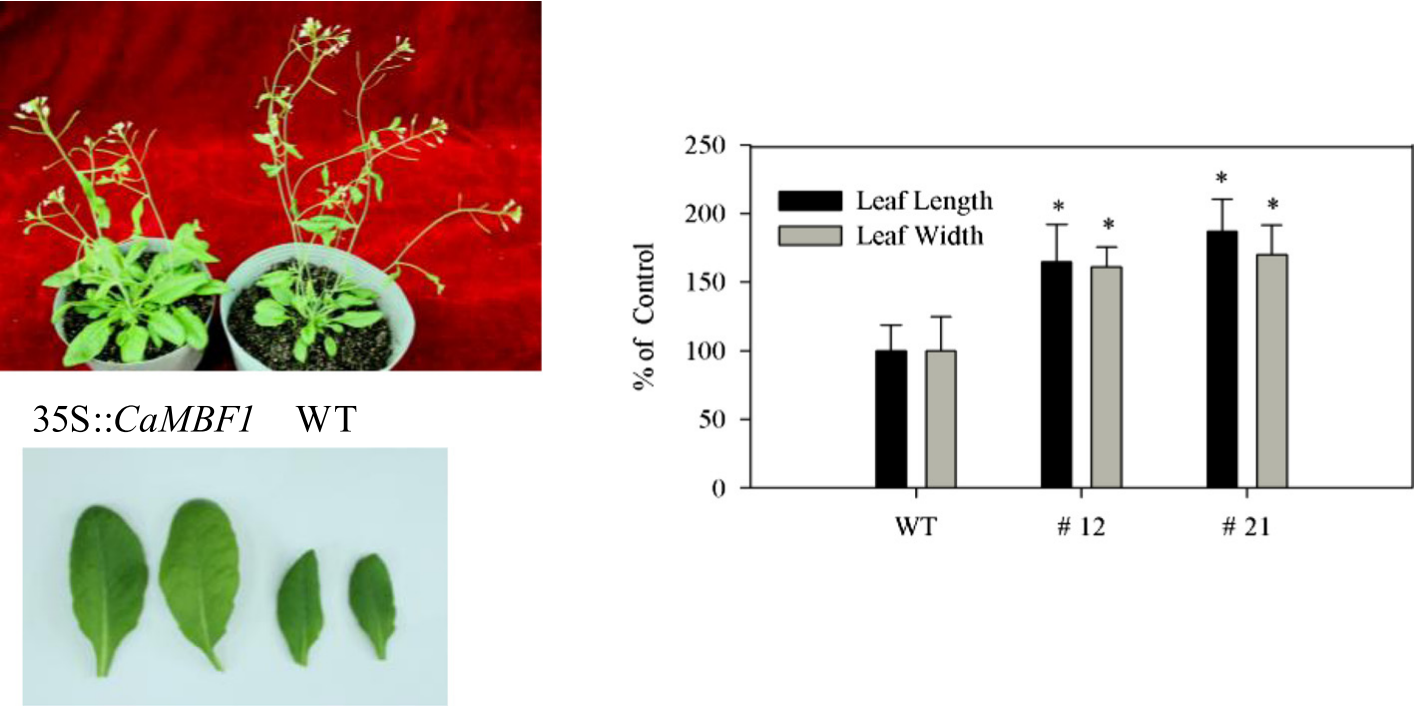
**Phenotypic analysis of wild-type and *CaMBF1*-overexpressing transgenic *Arabidopsis *(#12 and # 21).** Wild-type (Col-0) and transgenic *Arabidopsis* were grown at 22°C, with a 14/10 h photoperiod, a light intensity of 120 mmol m^−2^ s^−1^, and 70% relative humidity. *indicates the least significant difference (LSD) test significant at P < 0.05.

To test the function of *CaMBF1* in *Arabidopsis*, we generated transgenic plants that constitutively expressed *CaMBF1* under the control of the *CaMV 35S* promoter. Transgenic plants expressing *CaMBF1* appeared similar in their growth and development to WT plants. However, as shown in Figure 4, the transgenic plants were larger than the WT plants during the florescence production period; the rosette leaves of transgenic plants were 70% longer and 60% wider than those of WT plants.

**Figure 5:**
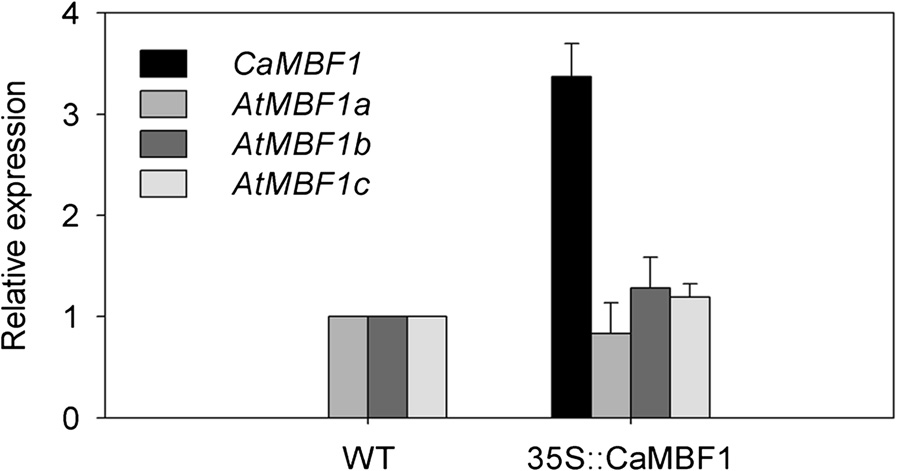
**Relative expressions of *AtMBF1s *transcripts in transgenic or wild-type plants under normal growth conditions.***Arabidopsis* encodes three different *AtMBF1* isoforms *(AtMBF1a,* At2g42680; *AtMBF1b*, At3g58680; *AtMBF1c*, At3g24500).

**Figure 6:**
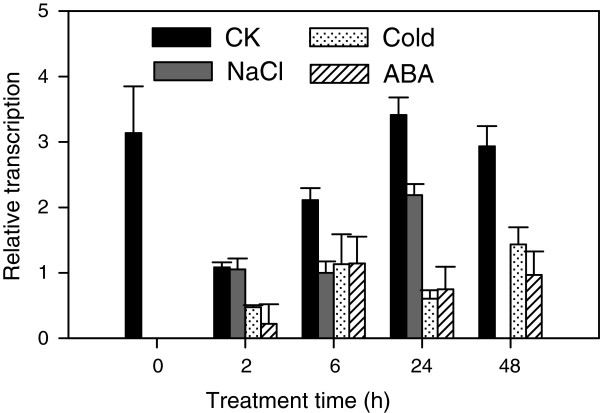
**Analysis of *CaMBF1 *expression profiles in transgenic lines in response to different stress treatments.** For salt stress and ABA treatments, 2-week-old seedlings were submerged in a MS/2 medium containing 150 mM NaCl and 100 μM ABA solutions, respectively. For cold treatment, 2-week-old transgenic seedlings were subjected to 4°C for 48 h. Samples were collected from both stress-treated and control (CK) plants at 0, 2, 6, 24, and 48 h of cold, salt stress and ABA treatment. *Arabidopsis eIF4A* gene (At3g13920) was used as an internal control for normalizing the variations in cDNA amounts used. Error bars represent standard error of means based on three independent reactions.

**Figure 7:**
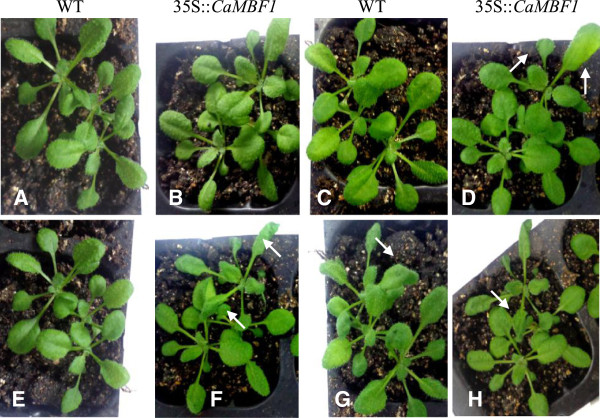
**Effect of cold stress on visual damage symptoms of wild-type and *CaMBF1*-overexpressing transgenic plants. A**, Wild-type *Arabidopsis* (Col-0) were subjected to cold stress for 2 h; **B**, Transgenic plants were subjected to cold stress for 2 h; **C**, Wild-type plants were subjected to cold stress for 6 h; **D**, Transgenic plants were subjected to cold stress for 6 h; **E**, Wild-type plants were subjected to cold stress for 24 h; **F**, Transgenic plants were subjected to cold stress for 24 h; **G**, Wild-type plants were subjected to cold stress for 48 h; **H**, Transgenic plants were subjected to cold stress for 48 h. The differences among treatments are marked with white arrows in rosette leaves. Photographs show plants subjected to cold stress for 48 h.

**Figure 8:**
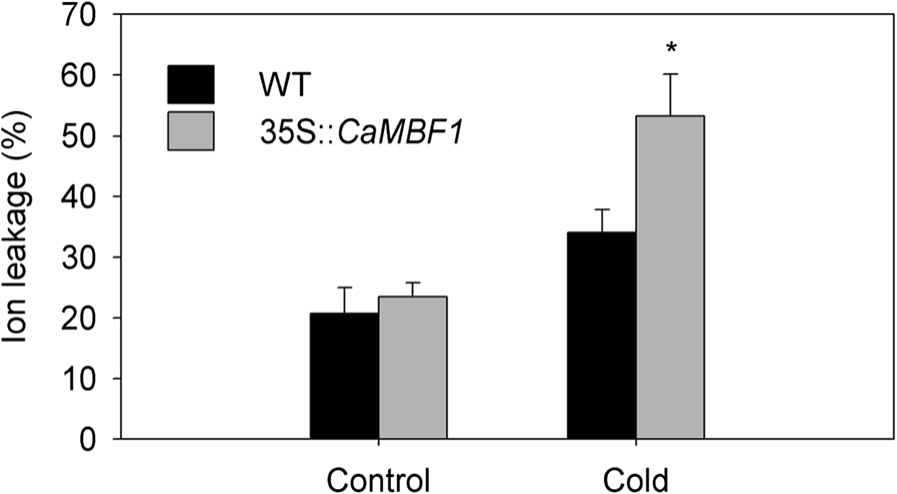
**Effect of cold stress on Electrolyte leakage of wild-type and *CaMBF1*-overexpressing transgenic plants.** 2-week-old WT and transgenic seedlings were exposed to low temperature 4°C for 24 h. Electrolyte leakage was expressed as a percentage of total electrolytes. Data are mean values (±SD) of at least three independent experiments. *indicates significantly different values between treatments (P < 0.05).

To study the response of *CaMBF1*-expressing plants to abiotic stress, 2-week-old WT and transgenic seedlings were subjected to several stresses, including cold, salinity, and ABA. Firstly, transcript levels of the high homology (*AtMBF1a*, *AtMBF1b* or *AtMBF1c*) modulated by the overexpression of *CaMBF1* under normal conditions were determined by qRT-PCR. Compared to WT plants, the expression of the homologous genes was not basically altered in transgenic plants when grown in normal condition (Figure 5), indicating that overexpression of pepper *CaMBF1* gene has no obvious effect on *AtMBF1s* transcripts in *Arabidopsis*. The *CaMBF1* gene was not detected in WT plants. *CaMBF1* transcript in transgenic plants subjected to cold stress, salinity, and ABA was much lower than that detected in transgenic plants under normal conditions (Figure 6), suggesting that expression of *CaMBF1* in *Arabidopsis* was dramatically decreased by stress treatments such as cold, salinity, and ABA. Furthermore, the visible symptoms of leaf damage in transgenic seedlings were observed to examine the tolerance of *CaMBF1*-expressing plants to cold stress. As shown in Figure 7, overexpression of the pepper *CaMBF1* gene in *Arabidopsis* aggravated the visible symptoms of leaf damage caused by cold stress in seedlings. Wilting appeared after 6 h of cold stress in transgenic plants and became serious at 24 h, while control leaves only exhibited withering after 48 h of cold stress. Meanwhile, to evaluate the extent of cell damage caused by cold stress in *CaMBF1*-expressing seedlings, electrolyte leakage was measured. The transgenic plants presented 1.5 folds higher electrolyte leakage than WT, which suggests that the membrane is likely to be impaired in these seedlings subjected to cold stress (Figure 8). These results suggested that overexpression of *CaMBF1* in *Arabidopsis* could downregulate the expression of genes involved in stress tolerance.

**Figure 9:**
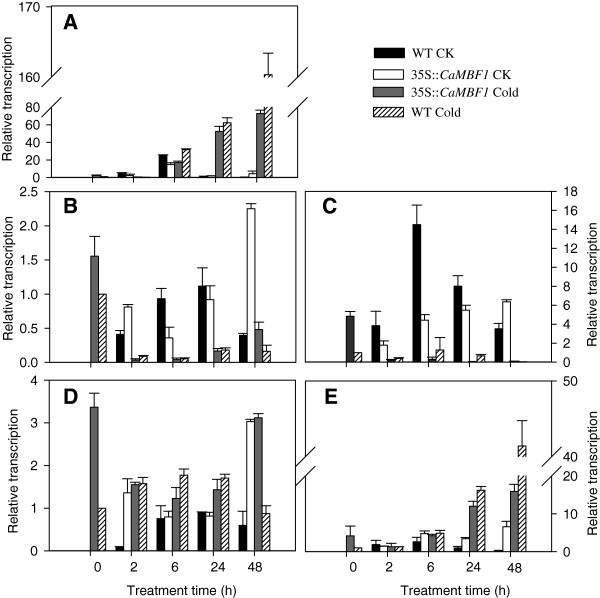
**Expression of stress-responsive genes in wild-type and transgenic plants subjected to cold stress.** Relative expression levels of stress-responsive genes were determined by qRT-PCR using cDNA synthesized from total RNAs isolated from the leaves of 2-week-old *Arabidopsis* exposed to low temperature 4°C for 48 h. **A**, *RD29A*; **B**, *RAB18*; **C**, *RD22*; **D**, *ERD15*; **E**, *KIN1.* There were four treatments: WT CK represents wild-type plants grown under non-stressed conditions; 35S::*CaMBF1* CK represents transgenic plants grown under non-stressed conditions; 35S::*CaMBF1* Cold represents transgenic plants subjected to cold stress; WT Cold represents wild-type plants subjected to cold stress. *Arabidopsis elF4A* gene (At3g13920) was used as an internal control for normalization of different cDNA samples. The expression levels of stress-responsive genes in wild-type plants at 0 h were used as control (quantities of calibrator) and were assumed as 1. Three biological triplicates were averaged and Bars indicate standard error of the mean.

We selected a group of candidate genes and conducted qRT-PCR analysis to test this hypothesis (Figure 9). Earlier studies have found *RD29A*, *RD22*, *RAB18*, *KIN1* and *ERD15* to be involved in the response to dehydration and cold/ABA [20-23]. Compared with normal conditions, cold stress induced *RD29A*, *ERD15* and *KIN1* genes expression in both transgenic and WT plants (Figure 9A, D and E). After cold treatment, the expression of *RD29A*, *ERD15* (except at 48 h) and *KIN1* in the transgenic plants was lower than that in the WT plants. Meanwhile, *RAB18* and *RD22* transcripts were dramatically decreased in both transgenic and control plants subjected to cold stress (Figure 9B and C). The expression of the *RD22* gene was basically not detected in transgenic plants under cold stress; the decrease in *RAB18* expression in transgenic plants was similar to that in WT plants during 24 h of cold stress. Overall, after cold treatment overexpression of the *CaMBF1* gene in *Arabidopsis* suppressed chilling-induced *RD29A*, *ERD15* and *KIN1* transcripts and aggravated chilling-decreased *RD22* expression. Therefore, *CaMBF1* appeared to act as a negative regulator of stress-responsive gene expression such as *RD29A*, *ERD15 KIN1* and *RD22*, consistent with the results from leaf chilling injury assays and electrolyte leakage measurement.

### The *CaMBF1*-overexpressing *Arabidopsis* is hypersensitive to salt stress

**Figure 10:**
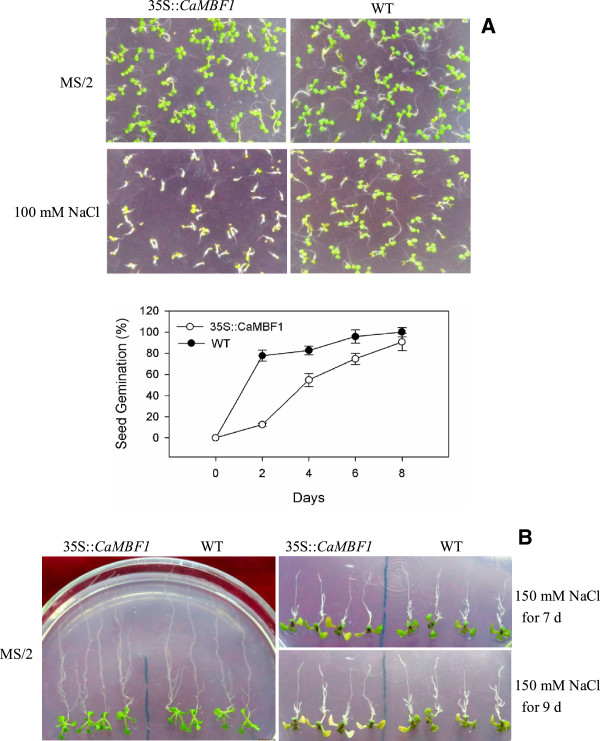
**Analysis of 35S::*CaMBF1 *transgenic lines subjected to salt stress. A**, Effects of salinity on germination. Complete radicle emergence was used as a marker for germination. 50 seeds were counted at indicated days, and Data represent means standard deviation of three independent experiments. **B**, Post-germination assay of transgenic seedlings. 6-day-old seedlings were transferred to half-strength Murashige and Skoog (MS/2) medium without (right panel) or with (left panel) 150 mM NaCl. Photographs were taken at 7 d or 9 d after the transfer.

To further characterize the tolerance of *CaMBF1*-overexpressing plants to salinity, transgenic seeds were germinated in MS/2 media supplemented with 100 mM NaCl and allowed to grow for 8 days. Transgenic seeds exhibited hypersensitivity to salinity compared with WT seeds (Figure 10A). On medium containing 100 mM NaCl, 78% of WT seeds germinated within 2 d, whereas the germination percentage for transgenic seeds was only 12% during the same period. In addition, the germination and subsequent growth of transgenic seedlings were comparable to WT plants on normal medium, but were significantly more inhibited by salt stress (Figure 10). The cotyledons of 6-day-old transgenic lines were bleached 7 days after transfer to medium containing 150 mM NaCl and became serious at 9 days, whereas the cotyledons of WT plants were slightly affected (Figure 10B). On the other hand, the primary root growth of transgenic plants was similar to that of WT plants under salt stress. However, lateral root formation was more severely influenced by salinity in transgenic plants compared with WT plants (Figure 10B).

**Figure 11:**
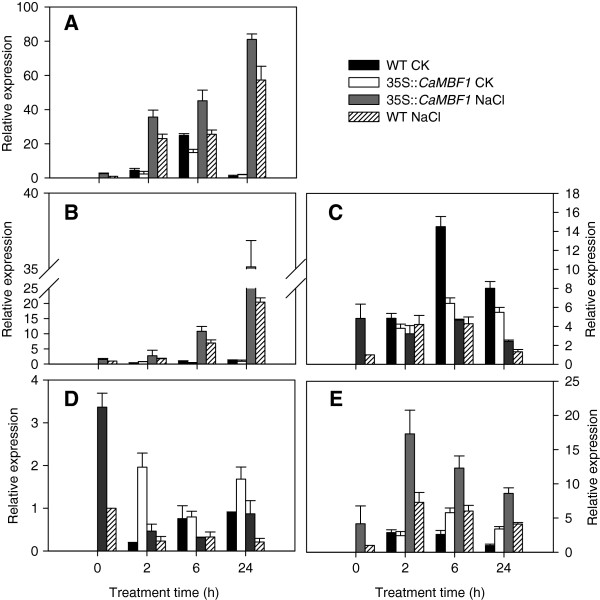
**Expression of stress-responsive genes in wild-type and transgenic plants subjected to salt stress.** Relative expression levels of stress-responsive genes were determined by qRT-PCR using cDNA synthesized from total RNAs isolated from the leaves of 2-week-old *Arabidopsis* subjected to high-salt stress (150 mM NaCl) for 24 h. **A**, *RD29A*; **B**, *RAB18*; **C**, *RD22*; **D**, *ERD15*; **E**, *KIN1.* There were four treatments: WT CK represents wild-type plants grown under non-stressed conditions; 35S::*CaMBF1* CK represents transgenic plants grown under non-stressed conditions; 35S::*CaMBF1* NaCl represents transgenic plants subjected to salt stress; WT NaCl represents wild-type plants subjected to salt stress. *Arabidopsis elF4A* gene (At3g13920) was used as an internal control for normalization of different cDNA samples. The expression levels of stress-responsive genes in wild-type plants at 0 h were used as control (quantities of calibrator) and were assumed as 1. Three biological triplicates were averaged and Bars indicate standard error of the mean.

Similarly, comparative expression analyses of the stress gene markers described above were also performed by qRT-PCR on RNA isolated from 2-week-old plants grown under non-stress and salt stress conditions (Figure 11). Upon salinity treatment, several gene markers (*RD29A*, *RAB18* and *KIN1*) were highly induced in both WT and transgenic seedlings (Figure 11A, B and E). Conversely, *RD22* and *ERD15* transcripts were dramatically decreased in both transgenic and WT plants subjected to salt stress (Figure 11C and D). Furthermore, the expression of *RD29A*, *RAB18*, *KIN1* and *ERD15* in the transgenic lines was higher than that in the WT plants under high salt conditions. Therefore, overexpression of *CaMBF1* in *Arabidopsis* appeared to positively regulate the expression of stress-responsive gene markers such as *RD29A*, *RAB18*, *KIN1* and *ERD15*, which was not consistent with the results from seed germination and cotyledon greening assays. In some cases, the level of stress gene expression appears to be insufficient to induce tolerance changes [24-26].

### Altered expression of stress-responsive *HSPs* in the *CaMBF1*-overexpressing *Arabidopsis*

**Figure 12:**
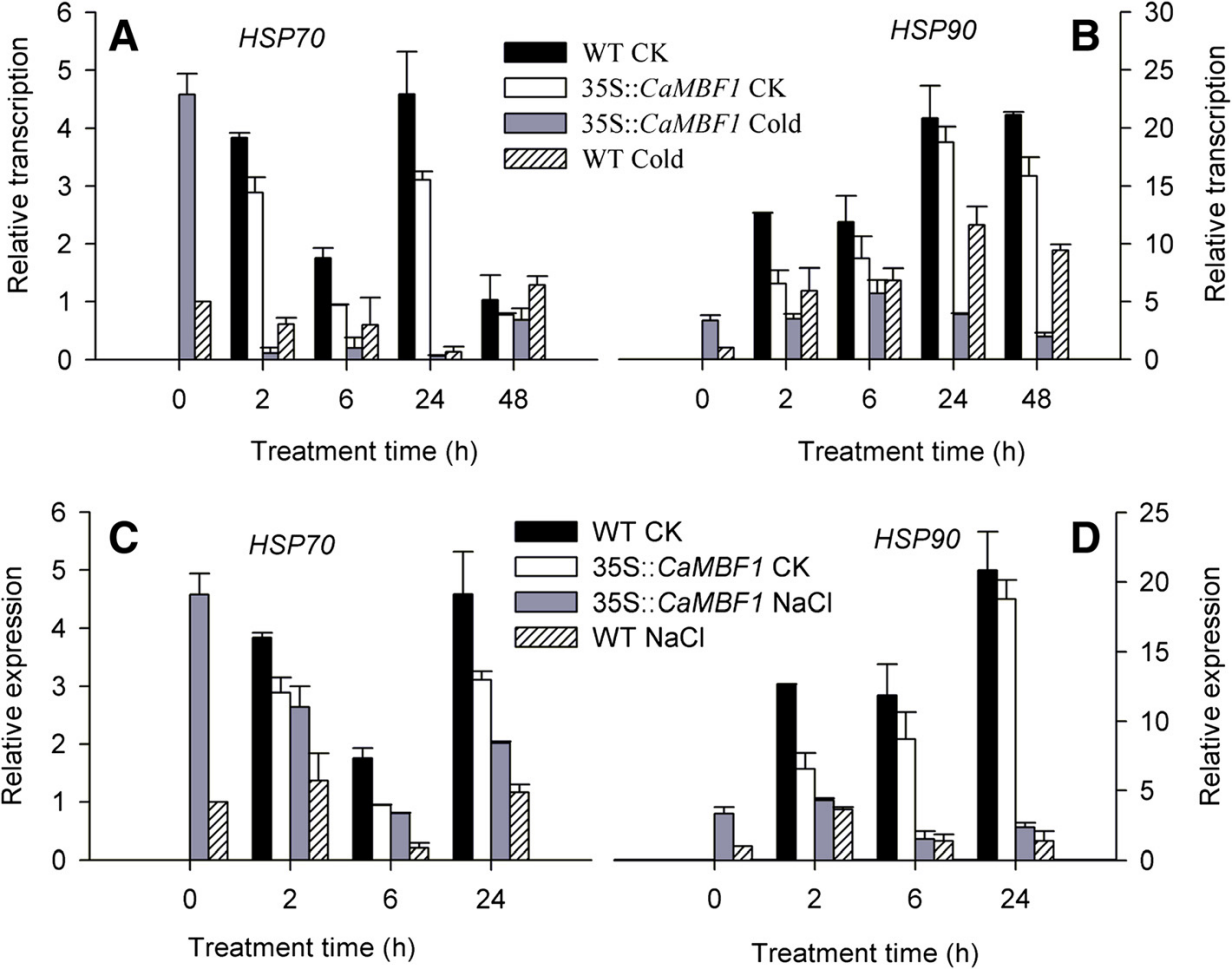
**Expression of *HSPs *in wild-type and transgenic plants subjected to cold and salt stresses.** Relative expression levels of stress-responsive genes were determined by qRT-PCR using cDNA synthesized from total RNAs isolated from the leaves of 2-week-old *Arabidopsis* subjected to cold stress for 48 h and high-salt stress for 24 h as described above, respectively. **A**, *HSP70 from Arabidopsis* under cold stress; **B**, *HSP90 from Arabidopsis* under cold stress; **C**, *HSP70 from Arabidopsis* under salt stress; **D**, *HSP90 from Arabidopsis* under salt stress*.*

To evaluate whether *CaMBF1* expression could be correlated with alterations of other stress-responsive genes, classical heat-shock genes, *HSP70* and *HSP90* were tested in all lines by qRT-PCR (Figure 12). Compared with control plants, *HSP70* and *HSP90* transcripts (except at 0 h) were decreased in transgenic plants under normal conditions. After cold treatment, the expression of *HSP70* and *HSP90* in the transgenic plants was lower than that in the WT plants (Figure 12A and B); whereas, the expression of these genes in the transgenic lines was higher than that in the WT plants under high salt conditions (Figure 12C and D), indicating that comparative regulation of *HSPs* in response to *CaMBF1* overexpression could be related to different stresses.

## Discussion

Here, we report a putative transcription coactivator from pepper seedlings, the putative amino acid sequence of which was 95% and 80% identical to those of *StMBF1* and *AtMBF1b*, respectively. Therefore, *CaMBF1* could be categorized as belonging to the same group as *StMBF1*[8]. The deduced amino acid sequences of plant *MBF1s* revealed the existence of highly conserved amino acid residues in each group [19]. Additionally, tissue-specific expression of *CaMBF1* observed here (Figure 2) suggests that *CaMBF1* may be involved in physiological processes of pepper plants. In this regard, the highly homologous *StMBF1* also exhibits a ubiquitous tissue distribution [8].

In the present study, *CaMBF1* transcript in pepper or *Arabidopsis* seedlings was dramatically decreased in response to abiotic stresses such as SA, ABA, high salt, osmotic, and heavy metal stress (Figures 3 and 6). Particularly, under cold stress the expression of *CaMBF1* was downregulated in *Arabidopsis* seedlings (Figure 6). These results indicated that *CaMBF1* may be negatively involved in stress signaling pathways. Unlike other *MBF1* genes, the expression of *AtMBF1c* is induced by various stresses, including salinity, drought, heat, H_2_O_2_ and ABA, and is not affected by cold stress [19]. Salinity also induced *AtMBF1a/b* expression [10] and cold stress did not significantly change mRNA accumulation of *AtMBF1a* and *AtMBF1b* in *Arabidopsis*[19].

*CaMBF1*-overexpressing plants showed extremely large leaf phenotypes (Figure 4). This finding could be explained by similar evidence reported by Tojo *et al.*[27] who suggested that *AtMBF1*s play a crucial role in controlling rapid leaf expansion through promotion of cell expansion. The amino acid sequences of *MBF1*s are widely conserved among plant species. Similarly, transgenic *Arabidopsis* expressing *AtMBF1c* were 20% larger than control plants and produced more seeds [9].

The visible symptoms of leaf damage in *CaMBF1*-expressing transgenic *Arabidopsis* were observed more severely than that in WT plants (Figure 7) and the transgenic plants presented 1.5 folds higher electrolyte leakage than WT under cold stress (Figure 8), suggesting that the tolerance of transgenic plants to cold stress was reduced. This result was in agreement with the fact that some genes isolated from the reverse SSH library, including a *MBF1* homologue, were related to reduction in cold tolerance of plants [17]. Moreover, overexpression of the *CaMBF1* gene in *Arabidopsis* reduced the expression of *RD29A*, *ERD15*, *KIN1*, and *RD22* during cold treatment (Figure 9). *CaMBF1* may reduce the tolerance of *Arabidopsis* to cold stress by negatively regulating stress-tolerant gene expression. Suzuki *et al.*[9] reported that the tolerance of *MBF1c*-expressing transgenic seedlings to cold stress was similar to that of WT seedlings. On the other hand, *CaMBF1*-expressing transgenic plants showed high susceptibility to salt stress imposed during seed germination (Figure 10A). In contrast to this result, the triple knock-down mutant (*abc-*) presented a significant diminution of germination under osmotic stress [28] and *MBF1* genes negatively regulated ABA-dependent inhibition of germination [29]. The cotyledons and lateral root formation were more severely influenced by salinity in transgenic plants compared with WT plants (Figure 10B). Meanwhile, root growth of MBF1a/c-expressing plants adopted to the high or low-salt condition comparatively better than WT plants [9,10]. Seed germination is controlled by the antagonistic action of gibberellic acid (GA) or ethylene and ABA [30-32]. *MBF1* may be involved in several hormone signal transduction pathways (ethylene, GA/ABA) during seed germination [6,33]. In addition, the expression of *RD29A*, *RAB18*, *KIN1* and *ERD15* in *CaMBF1*-expressing transgenic *Arabidopsis* was higher than that in WT plants under high salt conditions (Figure 11). Kim *et al.*[10] also reported that *MBF1a*-overexpressing transgenic *Arabidopsis* induced *RD29A*, *ERD15*, and *KIN2* during the course of salt treatment. The accumulation of a number of defense transcripts was similarly augmented in *MBF1c* transgenic *Arabidopsis* in response to heat stress [9].

The expression patterns of the above-mentioned stress gene markers in transgenic plants subjected to cold stress were different from those in transgenic lines under salt stress. This difference could be related to that each stress opens out specific defense mechanisms in young seedlings and the participation of *CaMBF1* might be different depending on the stress condition imposed. Since different stresses may disrupt plant growth and development in specific ways, the plant might alleviate damage by different mechanisms. The results of this study, that overexpression of the pepper *CaMBF1* gene differently modules the expression of *HSPs* in *Arabidopsis* under cold and salt stresses (Figure 12), supported this hypothesis. There were similar reports as follows: constitutive expression of stress-responsive HSP genes was augmented in the *abc-* mutant, indicating that AtMBF1s may act as negative regulators of HSP in *Arabidopsis thaliana* seedlings [28]. Suzuki *et al.*[9] described that transcripts encoding classical HSPs accumulated to a similar level in WT and transgenic plants over-expressing *MBF1c*; they suggested that the enhanced tolerance of these plants to osmotic and heat-shock stress was associated with the expression of other stress-responsive genes rather than with the constitutive expression of HSPs. Finally, our data together with previous evidences support that *Capsicum annum CaMBF1* play a different role as *Arabidopsis AtMBF1* in response to salt or cold stress. Further studies will be necessary to reveal specific functions for each gene.

## Conclusions

This study demonstrates that the manipulation of the *CaMBF1* gene from pepper using a transgenic approach can lead to reduced cold-stress and salt-stress tolerance in *Arabidopsis*. In addition, overexpression of *CaMBF1* may reduce stress tolerance by downregulating stress-responsive genes to aggravate the leaf damage caused by cold stress. However, upregulation of such stress-responsive genes appears to be insufficient to induce tolerance of *CaMBF1* transgenic plants to salt stress. The *CaMBF1* gene could be a candidate gene for future research on abiotic stress signaling pathways and genetic engineering of novel pepper cultivars. The results of this study will be helpful in providing beneficial information to support biotechnology applications and molecular breeding, which clarify the function of a gene involved in abiotic stress in plants.

## Methods

### Plant materials and stress treatments

Pepper (*Capsicum annuum* L.) cv. P70 seeds were sown at a depth of 1.0 cm into 9-cm-deep plastic pots filled with growth medium consisting of grass charcoal and perlite in a ratio of 3:1 after accelerated germination and grown in a growth chamber using a previously described method [16]. The seedlings at the sixth leaf expansion stage were used to establish the following treatments. ABA and cold treatments were performed as described by Guo *et al.*[17]. For ABA and cold treatments, seedlings were sprayed with freshly prepared 0.57 mM ABA solution or water (control). At 72 h after foliar application, control and ABA treatment groups were subjected to chilling stress at 6°C. For salt, osmotic, and the heavy metal (Hg) treatments, the seedling roots were immersed in solutions containing 300 mM sodium chloride (NaCl), 300 mM mannitol, or 300 μM Hg and maintained at 25°C for the indicated times. For SA treatment, seedlings were sprayed with 5 mM SA solution and incubated for the indicated times. The treated seedlings were harvested after 0, 1, 3, 6, 12 and 24 h for examination of *CaMBF1* expression pattern under various stress conditions. At each time point, two or three upper young leaves from four separate seedlings were collected to form one sample, wrapped with foil, immediately frozen in liquid nitrogen and stored at −80°C. The treatments were arranged in a randomized complete block design with three replicates.

### Isolation of *CaMBF1* cDNA clone and sequence analysis

The *MBF1*-homologous EST (GenBank No: JZ198811) characterized from the differential screening of a cold-related pepper seedling cDNA library was reported by Guo *et al.*[17]. The full-length open reading frame of the *MBF1* homologue was obtained using the cDNA fragment of this homolog as a probe by a homology-based candidate gene method [34]. The full-length forward and reverse primers for *CaMBF1* were 5′-GAAGAAAAAAAGCAATGAGTGG-3′ and 5′-GCAGAAACGAATTTAG-GATTTG-3′ respectively. The theoretical molecular weight (Mw) and isoelectric point (pI) were calculated with the ExPASy compute pI/Mw tool [35]. Sequence data were analyzed using Clustal W [36]. Homology searches in database were carried out using the default parameters of the BLAST program on the website http://www.ncbi.nlm.nih.gov:blast[37].

### Generation of *CaMBF1* transgenic *Arabidopsis* plants

Full-length forward and reverse primers with an added *Bam*HI site were used to generate a DNA fragment encoding the *CaMBF1* gene. The *CaMBF1* fragment was inserted into the cloning site of the pMD19 T-vector (Takara, Tokyo, Japan) and then this plasmid DNA was digested using *Xba*I and *Bam*HI from the pMD19 T-vector. The *CaMBF1* DNA fragment was inserted into the *Xba*I-*Bam*HI site of the pVBG2307 vector under the control of the 35S cauliflower mosaic virus (CaMV) promoter, resulting in the pVBG2307-*CaMBF1* construct. The pVBG2307 vector was constructed according to pCAMBIA2300 vector [38]. This construct was confirmed by sequencing and then introduced into *Agrobacterium tumefaciens* GV3101 using electroporation. *Arabidopsis* (ecotype Columbia-0, Col-0), chosen for transgenic studies, was grown in a controlled environment chamber at 22°C, with a 14/10 h photoperiod, a light intensity of 120 mmol m^−2^ s^−1^, and 70% relative humidity. Transgenic plants were generated by *Agrobacterium*-mediated transformation using the floral dip method [39]. *CaMBF1*-overexpressing transgenic seedlings were confirmed by examining the segregation ratio of the kanamycin selectable marker and by PCR analysis of *NPTII* and *CaMBF1* using the primers *NPTII*-F/R and *CaMBF1*-F/R (Additional file 1: Table S1). T2 lines that produced 100% kanamycin -resistant plants in the T3 generation were considered as homozygous transformants. In each experiment, T2 generations of homozygous transgenic lines (#5, #12 and #21) were selected for further analysis. Similar phenotypes and results used for this study were observed in more than three independent lines of transgenic plants.

### Performance of transgenic lines under stress treatments

Two-week-old transgenic seedlings were subjected to various treatments. Cold treatment was conducted in the dark by exposure of plants grown on vermiculite soil at 22°C to 4°C for 48 h, whereas control plants were placed in the dark at 22°C for 48 h. After cold treatment, wild-type (WT) and *CaMBF1*-overexpressing transgenic plants were visually examined to determine the extent of chilling damage. For high-salinity and ABA treatments, 2-week-old seedlings were submerged in half-strength Murashige and Skoog (MS/2) medium containing 150 mM NaCl or 100 μM ABA solutions, whereas control plants were submerged in a MS/2 medium. Third–fourth rosette leaves were collected from both stress-treated and control plants after 0, 2, 6, 24, and 48 h of cold, salt stress or ABA treatments. At each time point, sample was frozen in liquid nitrogen, stored at −80°C and used for extraction of total RNA. The treatments were arranged in a randomized complete block design with three replicates.

Homozygous T2 seeds of the transgenic lines were used for phenotypic analysis. For high-salinity treatment, seeds of WT and transgenic plants were plated on MS/2 agar plates supplemented with 100 mM NaCl, grown in a growth chamber, and assessed for percentage of germination after various times (0, 2, 4, 6 and 8 d). Experiments were done in triplicate for each line (50 seeds each). 6-day-old plants grown on normal MS/2 agar plates were transferred to vertical MS/2 agar plates containing 150 mM NaCl, and grown for another week as previously described [40]. The root growth and cotyledon greening of 24 seedlings were observed.

### Measurement of electrolyte leakage

Leaflets from 2-week-old seedlings were transferred to 4°C and incubated for 24 h in the dark in the growth chamber. The conductivity of the suspending solution was measured according to the method of Arce *et al.*[28]. The electrical conductivity of the solution was measured using an electrical conductivity analyzer (DDS-307; Shanghai Precision Scientific Instrument Co., Ltd., China) before and after autoclaving at 120°C for 30 min to release the total electrolytes. The conductivity was scored at least for 4 plants per line and pretreatment. Electrolyte leakage was expressed as a percentage of total electrolytes.

### Real-time quantitative PCR (qRT-PCR) analysis

RNA extraction, cDNA preparation and qRT-PCR were performed as described by Guo *et al.*[17]. Relative gene expression levels were determined using the 2^-ΔΔ CT method. Total RNA was extracted from the leaves of pepper plants subjected to various stress for 0, 1, 3, 6, 12, and 24 h as described above. The ubiquitin -conjugating protein gene (*UBI-3*, GenBank accession no. AY486137.1) from pepper plants was amplified as a reference gene for normalization of *CaMBF1* cDNA samples. On the other hand, total RNA of *CaMBF1* transgenic and WT *Arabidopsis* were used to examine the expression of seven stress-related genes (*RD29A*, *RAB18*, *ERD15*, *KIN1*, *RD22*, *HSP70* and *HSP90*) and three *Arabidopsis* isoforms (*AtMBF1a, AtMBF1b, AtMBF1c*). *Arabidopsis eIF4A* gene (At3g13920) was included in the assays as an internal control for normalizing the variations in cDNA amounts used [41]. The corresponding specific primers were listed in Additional file 1: Table S1.

### Statistical analysis

Data were analyzed using analysis of variance (SAS 8.2, North Carolina State University, USA) and mean separation was analyzed using the least significant difference. The P value <0.05 was considered to be significant.

### Supporting data

All the supporting data are included as additional files.

## Competing interests

The authors declare that they have no competing interests.

## Authors’ contributions

WLG, RGC and ZHG conceived and designed the experiments; WLG, XHD, ZZ, YXY and GYW performed the experiments; ZZ and YXY analyzed the data; ZHG contributed reagents/materials/analysis tools; WLG wrote the paper. All authors read and approved the final manuscript.

## Supplementary Material

Additional file 1: Table S1 The sequences of primers used in this study. Click here for file
