# Supplementary material for: Reduced tolerance to abiotic stress in transgenic Arabidopsis overexpressing a Capsicum annuum multiprotein bridging factor 1
Source: BMC Plant Biol. 2014 May 20;14:138. doi: 10.1186/1471-2229-14-138 (PMC4047556; doi:10.1186/1471-2229-14-138)
Supplement: Additional file 1: Table S1 — The sequences of primers used in this study. [file 1471-2229-14-138-S1.doc]

Additional Table 1 The sequence of primers employed in this study

| Gene | Accession NO. | Forward (F) and Reverse (R) primer  5’→3’ |
| --- | --- | --- |
| *AtRD22*  *AtRD29A*  *AtRAB18*  *AtERD15*  *AtKIN1*  *AtMBF1a*  *AtMBF1b*  *AtMBF1c*  *HSP70*  *HSP90*  *eIF4A*  *UBI-3*  *CaMBF1*  *NPTII* | At5g25610  At5g52310  At5g66400  At2g41430  At5g15960.1  At2g42680  At3g58680  At3g24500  At3g12580  At5g52640  At3g13920  AY486137.1  JX402927 | F: GCGTTGGCAGCGGAAAA  R: GCGTTAGGATCGTCGTGG  F: CTGATCCCACCAAAGAAGAAACT  R: AAGCCCATCGGAGAATTC  F: AAGAAGAACATGGCGTCTTACC  R: GTTCCAAAGCCTTCAGTCCC  F: CCAGCGAAATGGGGAAAC  R: ACAAAGGTACAGTGGTGGC  F: AAATGTCAGAGACCAACAAGAA  R: CTACTTGTTCAGGCCGGTCTT  F: ACTGATGTAGCAAGTAACAAGAATC  R: CAACTATGTGATGAAAAGACC  F: AAGTGTAGAACAAAGCTCTTAAAG  R: ATAATGACAAAAGGTTCCAAACAGC  F: TGTTCCTTTCTCTCAATTCATCG  R: CATTTATCAAACAAAACAACAAGAC  F: GGCTGAGGCAGATGAGTTCGAGGA  R: GGCCAGCACCGCCGCTACCA  F: AAAGAAGAAGAAAATCAAGGAAGT  R: ACTGAGAAGTGTTTCACGGCTAA  F: TGACCACACAGTCTCTGCAA  R: ACCAGGGAGACTTGTTGGAC  F: TGTCCATCTGCTCTCTGTTG  R: CACCCCAAGCACAATAAGAC  F: TACATCTTTGGACACCAGGAA  R: GCAGAAACGAATTTAGGATTTG  F: AGACAATCGGCTGCTCTGAT  R: TCATTTCGAACCCCAGAGTC |
